# Unexpected posterior mediastinal mass: A case report and literature review

**DOI:** 10.1016/j.ijscr.2024.110544

**Published:** 2024-10-30

**Authors:** Messaoudi Houssem, Bessrour Habib, Raghmoun Wafa, Dardour Syrine, Mansouri Nada, Hachicha Saber

**Affiliations:** a Department of Cardiothoracic Surgery, The Principal Military Hospital of Instruction of Tunis, Tunisia; b Department of Cardiology, The Hospital of Manzel Bourguiba, Tunisia; c Department of Pathology, The Principal Military Hospital of Instruction of Tunis, Tunisia

**Keywords:** Glomangioma, Posterior mediastinum, Surgery

## Abstract

**Introduction:**

Glomangioma is an uncommon hypervascular tumor typically found in the extremities, with primary occurrences in the mediastinum being exceedingly rare.

**Case presentation:**

A 38-year-old male presented to our cardio-thoracic surgery department with chest pain. Chest computed tomography (CT) revealed a 45 mm posterior paravertebral right solidocystic mass, a finding confirmed by Magnetic resonance imaging (MRI) with a solidocystic mass in the posterior paravertebral right pleura suggesting a fibrous tumor exhibiting cystic degeneration. Surgical intervention under right thoracotomy involved the complete resection of the tumor. Postoperative analysis confirmed the diagnosis of a glomus tumor.

**Discussion:**

Glomus tumors are uncommon growths that arise from neuromyoarterial glomus bodies, which are specialized structures involved in regulating blood flow and skin surface temperature through arteriovenous connections. Our case represents the ninth instance of a glomus tumor in the mediastinum.

The clinical manifestation of intrathoracic glomus tumors lacks specificity and can vary depending on the location and size of the tumor, often resembling typical thoracic tumors.

Surgical resection remains the foremost approach for diagnosing and treating thoracic glomus tumors. Given the potential for malignancy, a radical resection is advised to ensure complete removal with clear margins.

**Conclusion:**

This case describes an uncommon presentation of a benign glomus tumor in the posterior mediastinum, highlighting its exceptional localization and emphasizing the critical importance of surgical resection for optimal management.

## Highlights

• Glomangiomas are rare, benign vascular tumors, typically occurring in the extremities.
• Mediastinal glomangiomas represent an exceedingly rare presentation.
• Surgical resection is essential for both diagnosis and treatment.
• Histopathological and immunohistochemical analysis are key to confirming the diagnosis of glomangioma.

## 1 Introduction and importance

The glomus, a neuro-arterial structure, can give rise to a hypervascular tumor termed glomangioma, characterized by modified smooth muscle cells within the glomus body that play a crucial role in regulating skin circulation. Typically found in the deep dermis of extremities and the subungual region of hands [1], glomus tumors are uncommon in the chest, the mediastinal presentation is exceedingly rare, with only nine examples reported in the literatures.

This study aims to provide a detailed presentation of a glomangioma identified in the right posterior mediastinal cavity, shedding light on this atypical manifestation and contributing to our understanding of primary glomus tumors in unusual anatomical locations.

This work has been reported in line with the SCARE criteria [2].

## 2 Case presentation

A 38-year-old man presenting with chest pain was admitted to our cardio-thoracic surgery department. He reported no fever, cough, or expectoration and his clinical and family history were unremarkable. Physical examinations revealed no abnormalities, and the chest X-ray was normal.

**Fig. 1:**
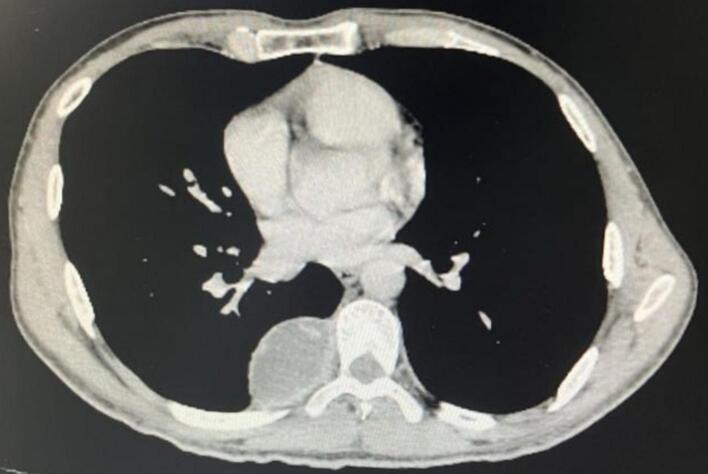
CT scan, mediastinal window showing the posterior mediastinal mass.

The CT scan revealed a 45 mm solidocystic mass in the posterior paravertebral right region, indicative of a fibrous necrotic tumor (Fig. 1).

**Fig. 2:**
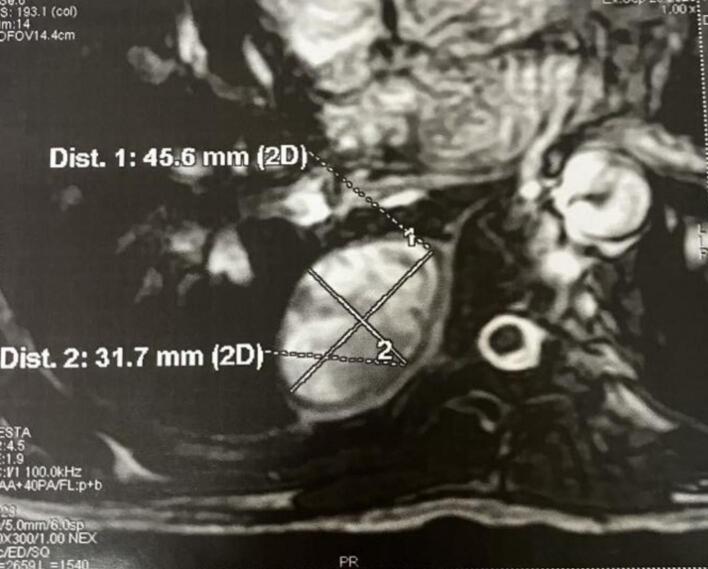
MRI showing the tumor to the right posterior mediastinum.

MRI demonstrated a posterior right paravertebral mass, which is solidocystic with a solid component showing hypointensity in both T1 and T2 signals, and enhancement after gadolinium injection. Its dimensions were 50 mm in the coronal plane and 45*30 mm in the transverse plane, suggesting a fibrous tumor undergoing cystic degeneration (Fig. 2). The mass came into contact with the posterior arches of the 8th and 9th right ribs and the vertebral bodies of D7, D8, and D9, with no osseous signal anomalies noted.

The patient underwent tumor resection. He was operated on under general anesthesia with selective intubation.

An initial thoracoscopy was performed, but due to the tumor's size (approximately 6 cm) and its close involvement with the spine, we opted to convert to an open thoracotomy for better access and control.

**Fig. 3:**
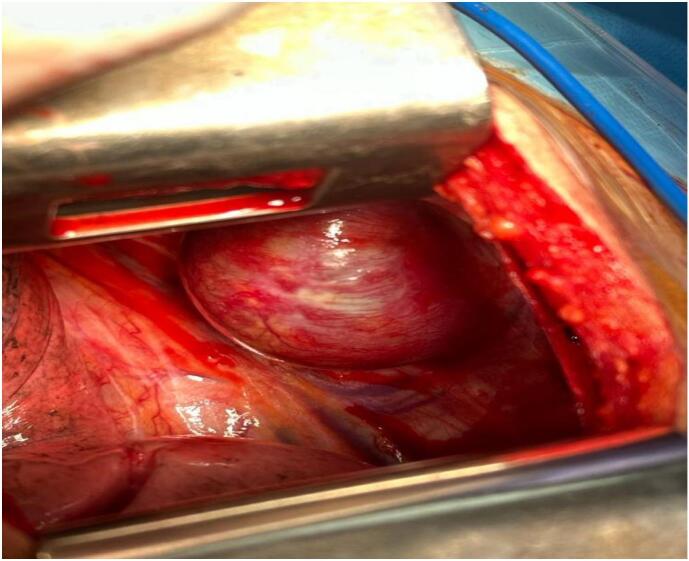
Per operative image of the tumor.

A right posterolateral thoracotomy was performed in the 6th intercostal space (Fig. 3).

The mass was completely resected, followed by closure over a chest tube. The postoperative course was uneventful, with removal of the tube on second postoperative day, and the patient was discharged the following day.

Macroscopically, the encapsulated mass measured 6*5*2.5 cm, exhibiting a multilocular and hemorrhagic appearance in the center with a solid peripheral component.

**Fig. 4:**
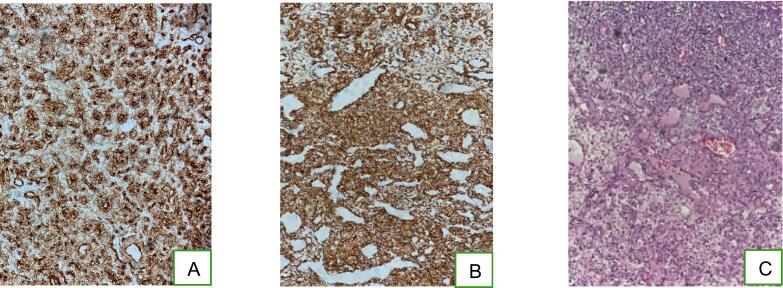
Microscopic findings. A: The tumor cells express smooth muscle actin (immunohistochemistry SMA*200). B: The tumor cells express CD34 (immunohistochemistry CD34*200) C: an upper contingent of round cells and a lower vascular contingent (hematoxylin and eosin).

Microscopic examination revealed a well-defined, benign proliferative tumor with a heterogeneous appearance, comprising solid clusters of round cells and a vascular component. The round cells formed solid sheets marked by a fine, branched hemangiopericytic vascularization. These cells were monomorphic with regular chromatin and showed no atypia or mitoses (Fig. 4).

Immunohistochemically, the tumor cells were positive for CD34 and AML, negative for CD31, synaptophysin, and PS 100, with no histological signs of malignancy.

In conclusion, the mediastinal mass exhibited histological features and an immunohistochemical profile consistent with a glomangioma.

## 3 Clinical discussion

Glomus tumors are uncommon growths that arise from neuromyoarterial glomus bodies, which are specialized structures involved in regulating blood flow and skin surface temperature through arteriovenous connections [3].

Accounting for 1.6 % of all soft tissue tumors, these tumors typically manifest in the upper extremities, predominantly in the subungual region of the fingers [4].

Our case represents the ninth instance of a glomus tumor in the mediastinum.

The first case was described by Brindley in 1949, located in the posterior mediastinum.

From 1949 to 2021, only 8 cases worldwide of glomus tumors in the mediastinum have been documented.

Table 1 represents the reported clinicopathologic data glomus tumor cases of mediastinum.

**Table 1:** Clinical and pathological data from documented cases of mediastinal glomus tumors.

Author	Age	Sex	Symptoms	Location	Size (cm)	Treatment	Diagnosis
Brindley [5]	29	F	Chest pain	Posterior	5	Resection	Glomus tumor
Choi et al. [6]	78	F	Dysphagia, dyspnea	Superior	4,5	Radiation	Malignant glomus tumor
Gaertner et al. [7]	46	F	Dyspnea	Superior	7	Resection	Glomus tumor
Bali et al. [8]	26	F	Back pain	Posterior, inferior	5	Resection	Atypical glomus tumor
Rychlik et al. [9]	59	M	Chest pain, abdominal pain	Posterior	2	Resection	Glomus tumor
Jang et al. [10]	21	F	No	Anterior inferior	5,3	Resection	Glomus tumor
Kanakis et al. [11]	69	M	No	Posterior	4,5	Resection	Glomus tumor
Fang et al. [12]	50	F	Cough	Posterior	2,7	Resection	Glomus tumor
Machboua et al. [13]	63	H	Hemothorax	Posterior		Interleukin alpha 2	Glomus tumor
Present case	38	H	Chest pain	Posterior	6	Resection	Glomus tumor

The clinical manifestation of intrathoracic glomus tumors lacks specificity and can vary depending on the location and size of the tumor, often resembling typical thoracic tumors.

Based on previous literature, the primary symptom is typically chest pain or dyspnea, but the tumor can be asymptomatic. In our case, the predominant symptom was chest pain, attributed to the tumor's chest wall involvement.

Radiographic findings are nonspecific, with X-ray or CT scans typically revealing a circumscribed soft tissue mass devoid of calcification. The rarity of intrathoracic glomus tumors can lead to confusion with other similar tumors.

Therefore, they are susceptible to misdiagnosis, highlighting the necessity of including mediastinal glomus tumors in the differential diagnosis of mediastinal neoplasms during evaluation.

Transthoracic thin-needle aspiration biopsy may contribute to preoperative diagnosis but is limited by small tissue samples.

Surgical resection remains the foremost approach for diagnosing and treating thoracic glomus tumors. Given the potential for malignancy, a radical resection is advised to ensure complete removal with clear margins.

Video-Assisted Thoracic Surgery (VATS) has become a widely adopted technique for the resection of small tumors in the posterior mediastinum, offering a minimally invasive approach with reduced postoperative complications and enhanced recovery outcomes compared to traditional open surgery [14].

Robotic-assisted surgery represents a promising for the resection of complex thoracic lesions. This advanced technique allows for greater precision, dexterity, and control due to its enhanced three-dimensional visualization and articulated instruments, which enable meticulous dissection in anatomically challenging regions such as the mediastinum [15].

While glomus tumors are generally benign, literature reports indicate malignant transformations [6].

Although morbidity and recurrence rates seem low in most cases, close follow-up is imperative.

Our patient was reviewed one month and six month post-operation, remains asymptomatic with no reported issues.

## 4 Conclusion

The rarity of glomus tumors in the mediastinum, combined with their nonspecific presentation, highlights the need to consider them in differential diagnoses of mediastinal masses. Surgical resection is essential for both diagnosis and treatment, with minimally invasive approaches offering effective options. Despite their generally benign nature, these tumors require careful follow-up due to potential recurrence or malignancy. Documentation of such cases is important to enhance clinical understanding and improve patient outcomes.

## Funding

This research did not receive any specific grant from funding agencies in the public, commercial, or not-for-profit sectors.

## Ethical approval

All procedures performed in studies involving human participants were by the ethical standards of the institutional and/or national research committee and with the 1964 Helsinki Declaration and its later amendments or comparable ethical standards. Ethical clearance was not necessary as the format of this paper is a case report.

## Consent

Written informed consent was obtained from the patient for publication and any accompanying images. A copy of the written consent is available for review by the Editor-in-Chief of this journal on request.

## Author contribution

Study concept: Dr. Messaoudi Houssem.

Manuscript writing: Dr. Bessrour Habib, Dr. Raghmoun Wafa.

Helped in Data interpretation and manuscript evaluation: Dr. Dardour Syrine Dr. Mansouri Nada.

Critical revision: Dr. Hachicha Saber.

## Guarantor

Bessrour Habib.

## Declaration of competing interest

Authors declare no conflict of interest.
